# Spaceflight on the ISS changed the skeletal muscle proteome of two astronauts

**DOI:** 10.1038/s41526-024-00406-3

**Published:** 2024-06-05

**Authors:** Marta Murgia, Jörn Rittweger, Carlo Reggiani, Roberto Bottinelli, Matthias Mann, Stefano Schiaffino, Marco V. Narici

**Affiliations:** 1 Department of Biomedical Sciences, University of Padova, 35131 Padua, Italy; 2 Max-Planck-Institute of Biochemistry, 82152 Martinsried, Germany; 3 Institute of Aerospace Medicine, German Aerospace Center, Cologne, Germany; 4 Department of Pediatrics and Adolescent Medicine, University Hospital Cologne, Cologne, Germany; 5 Science and Research Center Koper, Institute for Kinesiology Research, 6000 Koper, Slovenia; 6 Department of Molecular Medicine, University of Pavia, Pavia, Italy; 7 IRCCS Policlinico San Matteo Foundation, Pavia, Italy; 8 NNF Center for Protein Research, Faculty of Health Sciences, University of Copenhagen, Copenhagen, Denmark; 9 Veneto Institute of Molecular Medicine, 35129 Padua, Italy; 10 CIR-MYO Myology Center, 35121 Padua, Italy

**Keywords:** Physiology, Biochemistry

## Abstract

Skeletal muscle undergoes atrophy and loss of force during long space missions, when astronauts are persistently exposed to altered gravity and increased ionizing radiation. We previously carried out mass spectrometry-based proteomics from skeletal muscle biopsies of two astronauts, taken before and after a mission on the International Space Station. The experiments were part of an effort to find similarities between spaceflight and bed rest, a ground-based model of unloading, focused on proteins located at the costameres. We here extend the data analysis of the astronaut dataset and show compartment-resolved changes in the mitochondrial proteome, remodeling of the extracellular matrix and of the antioxidant response. The astronauts differed in their level of onboard physical exercise, which correlated with their respective preservation of muscle mass and force at landing in previous analyses. We show that the mitochondrial proteome downregulation during spaceflight, particularly the inner membrane and matrix, was dramatic for both astronauts. The expression of autophagy regulators and reactive oxygen species scavengers, however, showed partially opposite expression trends in the two subjects, possibly correlating with their level of onboard exercise. As mitochondria are primarily affected in many different tissues during spaceflight, we hypothesize that reactive oxygen species (ROS) rather than mechanical unloading per se could be the primary cause of skeletal muscle mitochondrial damage in space. Onboard physical exercise might have a strong direct effect on the prevention of muscle atrophy through mechanotransduction and a subsidiary effect on mitochondrial quality control, possibly through upregulation of autophagy and anti-oxidant responses.

## Introduction

During spaceflight, microgravity and ionizing radiation challenge the health and wellbeing of astronauts, who are also confronted with other inherent risks like high workloads, confinement, sleep disruption and exposure to higher levels of CO_2_ and continuous ambient noise^1,2^. One of the main goals of the ongoing research on the International Space Station (ISS), is to understand the mechanisms underlying the plethora of systemic multi-organ changes in astronauts^1,3,4^ as well as finding out how general biological processes are affected by the space environment in different organisms and cell systems^5–7^. This knowledge is necessary to develop effective countermeasures supporting human health in deep space missions.

Skeletal muscle, which makes up about 40% of human body mass^8^, undergoes extensive remodeling during spaceflight, leading to atrophy and loss of strength^2,9,10^. The decrease in leg muscle volume measured at landing after different missions is variable among crew members, approaching 40% in some individuals^10–12^. The percent loss of muscle force is typically larger than the corresponding decrease in size, as in other models of disuse and unloading^13^, pointing to general structural and functional rearrangements which affect force generation and transmission^14,15^.

Many effects of spaceflight on skeletal muscle can be reproduced by ground-based models of unloading, such as bed rest^10,16–18^, dry immersion^19^, unilateral limb suspension^20–22^ or orthotic unloading devices^23^ Studies in these models have shown that two days of unloading are sufficient to cause 2% reduction in the cross-sectional area (CSA) of vastus lateralis^24^. It has been shown that disuse atrophy is highly variable between muscles. In rats subjected to hindlimb unloading for fourteen days, plantar and soleus, showed more atrophy than tibialis anterior (TA) and extensor digitorum longus (EDL)^25^. The same duration of unilateral limb immobilization in humans caused a 4% reduction in the volume of medial gastrocnemius (MG) muscles but no change in TA, despite comparable fiber type composition^26^. These findings suggest that the results from one muscle cannot be easily extended to all others. In humans, antigravity postural muscles like soleus and vastus lateralis are strongly affected by unloading, both on Earth and in space^13,14^. Therefore, soleus muscle samples used in this study appear appropriate to monitor microgravity-dependent changes. Despite large individual variability in the response of the musculoskeletal system to unloading, contractile force typically showed even greater reduction, matching the observations in astronauts^13,27,28^. Different causes possibly contributed mechanistically to this phenomenon: (i) calcium signaling and excitation-contraction coupling were shown to be impaired by inactivity^28,29^; (ii) myosin expression was decreased during bed rest^30^; (iii) costameric protein complexes, involved in force transduction at the sarcolemma by connecting the Z disk of the sarcomere and the endomysium were downregulated by unloading^15,31^ and (iv) changes in neural function as well as neuromuscular instability, inducing the expression of neural cell adhesion molecule (NCAM), a marker of denervation, were observed in unilateral limb suspension and bed rest^28,32^. Exercise countermeasures during unloading on Earth, such as bouts of high-impact reactive jumps during 60 days of bed rest, completely preserved myofiber size^33^, leg muscle power and body composition^34^. During six months onboard of the ISS with exercise countermeasures, astronauts had lower loss of muscle CSA than after a short spaceflight of eleven days^35^. Altogether, these data indicate that loss of muscle mass and force in the absence of gravity might be mostly caused by the effects of unloading. However, the impact on muscle cells and the neuromuscular system of longer spaceflights, such as a mission to Mars or long-term sojourn on Lunar bases, involving extensive exposure to space radiation, is mostly unknown.

Large scale experiments carried out on the ISS with different model organisms and cultured cells, as well as in astronauts, have recently shown that mitochondria are severely affected by spaceflight^36,37^. In the NASA Twin Study, the astronaut twin showed reduced oxidative capacity and mitochondrial OXPHOS and fatty acid oxidation defects compared to the monozygotic twin serving as Earth-based control, leading to elevated level of circulating lipids during the mission^3^. Mass spectrometry-based proteomic comparison of muscle biopsies taken before and after short (eleven days, no countermeasures) and long (six months with exercise countermeasures) flights showed in both cases a general decrease of a subset of respiratory chain and tricarboxylic acid (TCA) cycle^35^. Aerobic metabolism appeared compromised post-flight in two astronauts who spent six months on the ISS irrespective of their very different training frequency and intensity^38^. Using single fiber proteomics, we have recently shown that ten days of bed rest caused changes in the expression of regulators of mitochondrial fission and fusion but no variation in abundance of respiratory chain subunits^39^.

The impact of disuse and unloading models on mitochondria and aerobic metabolism is controversial. Expression of the mitochondrial import receptor subunit TOM20 and of citrate synthase (CS), both widely used readouts for mitochondrial mass by immunoblot, did not show significant changes in young subjects during fourteen days of bed rest while their quadriceps volume decreased 6%^40,41^. In partial contrast, rotator cuff muscle of sheep after tenotomy showed mitochondrial dysfunction, which may be linked to the specific fatty degeneration propensity of the supraspinatus muscle^42^. It has been shown that there was little or no alteration of respiratory function in isolated fibers ex vivo at day 10 of bed rest^43^, whereas around 10% decrease in oxygen consumption was measured following 21 days of bed rest^44^. Other groups measured a decrease of skeletal muscle mitochondrial DNA/nuclear DNA content amounting to 15% after seven days of bed rest^45^. As susceptibility to muscle atrophy has been shown to differ greatly among different subjects^32,46^ and different muscles^25,26^, this might explain partially contrasting reports from different human cohorts of limited size and heterogeneous age. These data suggest that the decrease of muscle CSA caused by unloading can be accompanied by loss of mitochondria and oxidative capacity at Earth gravity, particularly at longer time points than ten days or in harsher unloading conditions, such as dry immersion^47^. However, data on short space flight in humans seem to indicate that the effects on mitochondrial downregulation may be much stronger and faster in the space environment^35^. Likewise, this suggests that mitochondrial dysfunction might have a slower and weaker mechanistic association to muscle atrophy at Earth gravity, whereas it is a hallmark of spaceflight in both mouse and human^36^.

To further investigate the controversial relationship between muscle disuse, mitochondria and aerobic metabolism, we here reanalyzed our proteomic dataset previously obtained from muscle biopsies of two astronauts who spent six months on the ISS and had very different levels of physical activity while on board^39^. Furthermore, a general proinflammatory state, including the upregulation of several inflammatory microRNAs and proteome modifications, had been detected at landing after the mission^48^. Building on technological advances, such as trapped ion mobility mass spectrometry (TIMS) with parallel accumulation-serial fragmentation (PASEF)^49^, we expanded the analysis of the skeletal muscle biopsies of these two astronauts, quantifying over 7100 proteins. We had limitedly used our previous analysis to shed light on the features of skeletal muscle remodeling that are common between bed rest and spaceflight^39^. The specific contribution of the present work is to enlarge the view of the effects of spaceflight on skeletal muscle with a hypothesis-free approach, through which mitochondria emerge as a pivot of change. The dataset covers 70% of all proteins annotated to the mitochondrion and reveals expression changes of proteins involved in the detoxification of reactive oxygen species (ROS) possibly linked to different levels of onboard exercise. Our analysis suggests a ROS-based mechanistic path for the development of countermeasures to preserve astronaut health.

## Results

### Experimental design and features of the dataset

**Fig. 1:**
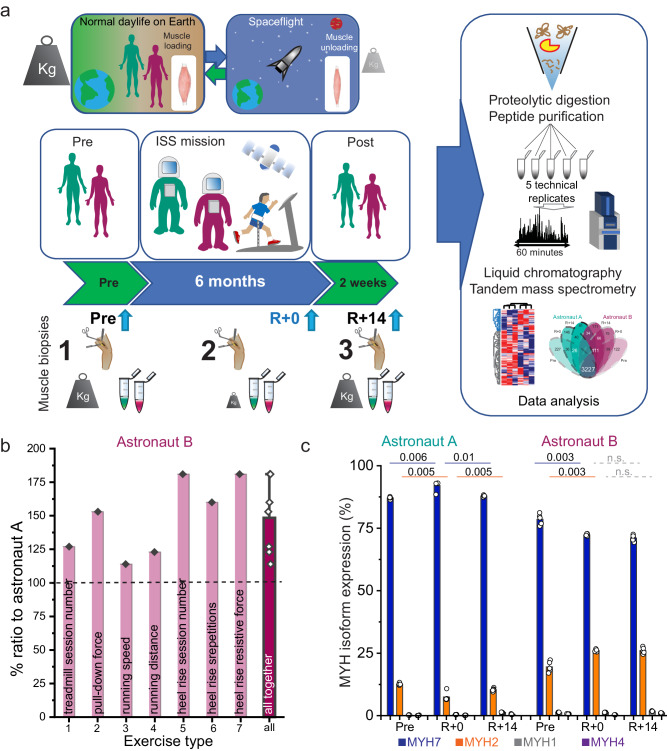
Study design and proteomic features of the dataset. **a** Timeline of muscle biopsies sampling, highlighting different body loading conditions during spaceflight and at earth gravity. This recapitulates the procedures used in our previous publication^39^. **b** Relative amount (ratio %) of onboard exercise training in astronaut B relative to astronaut A. The type of exercise is written within each bar. The last bar of the right shows all values combined, with standard deviation (*n* = 7). Data reanalyzed from our previous publication^38^. **c** Relative expression of four isoforms of myosin heavy chain (MYH) in the biopsies of the two astronauts at three timepoints. Pre, pre-mission; R + 0, day of landing; R + 14, two weeks after landing. Above the graph, the p value of two-sided paired *t* tests for MYH expression at different time points of the mission is shown, orange bars for MYH2 and blue bars for MYH7. Gray dotted bars, not significant (n.s.). Student *t* test, n = 5 technical replicates.

To assess how spaceflight modifies the structure and function of human skeletal muscle we analyzed by mass spectrometry-based proteomics a total of six soleus muscle biopsies from two astronauts who spent six months on the ISS^39^. Basal cardiorespiratory fitness as well as amount and intensity of physical activity performed on the onboard sport equipment were previously measured for each astronaut^38^. Our experimental design comprised a set of three biopsies per subject, one taken around 60 days before takeoff (Pre), one within 24 h of landing (R + 0) and one 14 days after landing (R + 14). The lysates were proteolytically digested and the purified peptides separated on a reverse-phase chromatography gradient prior to MS analysis on a Bruker timsTOF2 mass spectrometer, using parallel accumulation-serial fragmentation (PASEF) and data-dependent acquisition mode (Fig. 1a, see “Methods” for details). The peptides purified from each muscle biopsy were analyzed five times in the mass spectrometer (*n* = 5 technical replicates/sample, 30 samples in total). We quantified on average 3781 proteins per sample, ranging from 4342 to 3201 (Supplementary Fig. 1a). In all samples together, the analysis yielded 7192 proteins, spanning over seven orders of magnitude in intensity. Mitochondrial proteins were significantly enriched, together with sarcomere components, in the first of four intensity quartiles, whereas homeobox and zinc-finger proteins were enriched in the lowest intensity quartile, with ribosome biogenesis and mitochondrial translation annotation being prevalently distributed in the intermediate quartiles respectively (Supplementary Fig. 1b).

In our dataset, Pearson correlation between technical replicates of the same biopsy (*n* = 5) was consistently higher than between different muscle biopsies. The central diagonal squares, dashed yellow boxes, show the correlation between technical replicates, indicating high reproducibility of our MS workflow. Not only biopsies from the two astronauts but also those taken from the same subject at different timepoints (*n* = 3) had consistently lower Pearson correlation than technical replicates, particularly in the case of astronaut A. This highlights biological variability between individuals as well as between muscle biopsies sampled at different phases of the mission (Supplementary Fig. 1c). The median intensities of all quantified proteins showed minimal variation within each set of technical replicates and between different biopsies (Supplementary Fig. 1d). A core proteome quantified at least once in both astronauts (*n* = 6 biopsies) amounted to 5927 proteins, corresponding to 82% of total coverage (Supplementary Fig. 1e). The intersection of samples for individual timepoints of two astronauts shows that 45% of the total proteins in the dataset was quantified at least once in each sample (*N* = 30, Supplementary Fig. 1f). Over 55% of the proteins of each astronaut were quantified in the samples of all three timepoints (Supplementary Fig. 1g). The training regimen of the two astronauts differed regarding mechanical loading forces during their spaceflight on the ISS. They used a treadmill and an Advanced Resistive Exercise Device (aRED) to simulate the use of free weights in the absence of gravity. In all parameters analyzed by the Sarcolab study, astronauts B trained from 10% to over 80% more than astronaut A (Fig. 1b, data derived from Rittweger et al.^38^). We compared the expression of myosin heavy chain (MYH) isoforms in the six biopsies as a proxy to monitor fiber type shifts, which may have occurred during spaceflight as an effect of the change in muscle loading in the absence of gravity. The two astronauts had different basal composition of Myosin heavy Chain-7 (MYH7, type 1-slow) and MYH2 (fast-2A) in their soleus muscle, which showed small but significant changes at different phases of the mission (Fig. 1c).

### Changes in whole proteome of skeletal muscle in the two astronauts

To determine whether the total muscle lysates of different astronauts and mission time points could be distinguished based on proteomic features, we performed principal component analysis (PCA). This showed separation of the muscle proteomes from different time points of astronaut A along component 1, whereas the samples of astronaut B were largely overlapping with minor shifts (Supplementary Fig. 2a). The corresponding loadings suggested mitochondrial ATP synthase subunits and ECM proteins as drivers of component 1 (Supplementary Fig. 2b). This result shows that the proteomic difference between the two subjects may be larger than the overall longitudinal difference between time points of the mission. We thus performed PCA separately for the samples of each astronaut, obtaining a sharp separation of the biopsies sampled at the three time points along components 1 and 2 (Supplementary Fig. 2c, e). The corresponding major drivers are different for the two astronauts (Supplementary Fig. 2d, f). These findings prompted us to first compare biopsies taken at different time points and then focus our analysis on proteins and pathways that changed in both individuals.

**Fig. 2:**
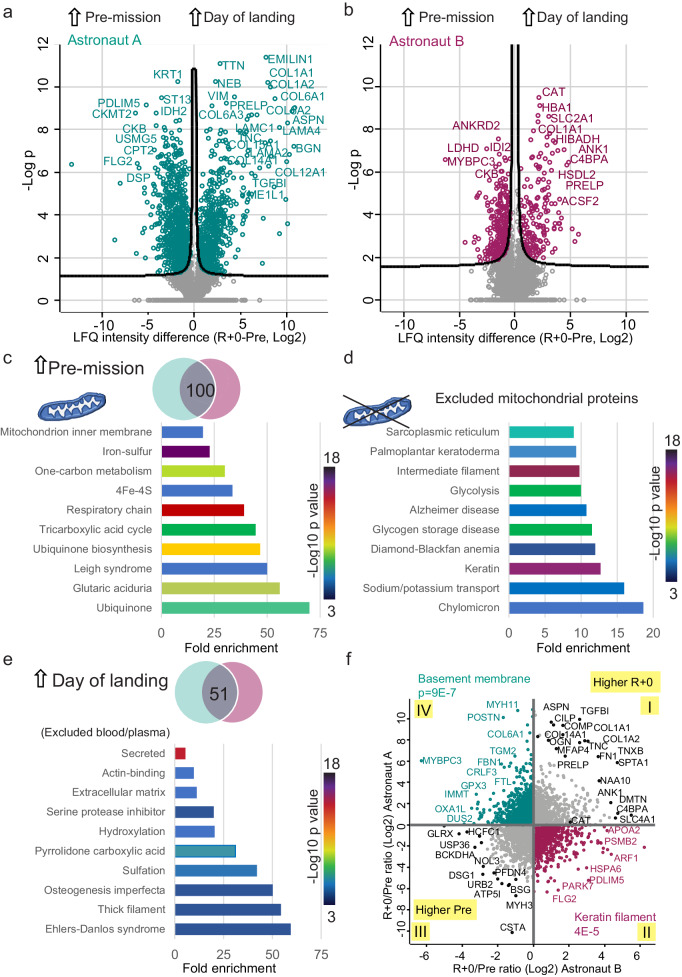
Effects of spaceflight on the skeletal muscle proteome. **a** Volcano plots comparing the muscle biopsy taken on the day of landing (R + 0) with the biopsy pre-flight (Pre) of astronaut A (*n* = 5 technical replicates). The gene name of a selected set of proteins with *p* value < 1E–5 is shown. Label free quantification (LFQ) intensity quantifies protein abundance (see “Methods”). **b** Same as A for astronaut B**. c** Top, Venn diagram showing the intersection of common proteins with significantly higher expression pre-mission in both astronauts (left sides of volcano plots for astronauts A and B respectively). Bottom, Keywords annotations most enriched among the 100 common proteins (background list, muscle proteome from Human Protein Atlas, 11756 entries in total). Fisher’s exact test, (FDR = 0.02). *P* value of the enrichment defines bar color, according to the scale on the right. **d** Ten Keywords annotations most enriched among common proteins, after excluding 796 mitochondrial proteins from the background list. **e** Top, Venn diagram showing the intersection of common proteins with significantly higher expression on the day of landing in both astronauts (right sides of volcano plots for astronauts A and B, respectively). Bottom, ten Keyword annotations most enriched among the 51 common proteins after excluding plasma and erythrocyte proteins from the background list. **f** Scatter plot comparing the Log2 expression ratio of proteins in the biopsy taken on the day of landing (R + 0) versus pre-flight (median of technical quintuplicates) of the two astronauts. Proteins commonly up- and down-regulated at R + 0 in both astronauts are marked with black symbols. Proteins upregulated at R + 0 in one astronaut but downregulated in the other (quadrants II and IV) have symbols color-coded for each astronaut. Gene names of proteins with largest changes are shown.

For astronaut A and B separately, we compared the biopsy taken pre mission with that taken on the day of landing, using volcano plots to pinpoint the proteins with significantly different expression (Fig. 2a, b and Supplementary Data 1 and 2). Intersecting the lists of significant hits in muscles pre-mission (left side of volcano plots), we found 100 proteins that were common to the two astronauts (Venn diagram, Fig. 2c, top). The ten annotations with highest fold enrichment in this protein list were all related to mitochondria. TCA cycle and respiratory chain were over fifty-fold enriched (Fig. 2c, bottom). Excluding mitochondrial proteins from the analysis, proteins with higher expression pre-mission were enriched in annotations related to cytoskeleton and cell adhesion (Palmoplantar keratoderma, intermediate filaments) as well as metabolism (chylomicron, glycolysis) (Fig. 2d). The biopsies taken on the day of landing showed a higher content of proteins related to blood and plasma than the samples pre-mission. We left out blood proteins from the analysis to focus on changes occurring in muscle. After this exclusion, biopsies post-mission from the two astronauts had 51 common significant hits (Fig. 2e, top) with specific enrichments in annotations related to the ECM and basement membrane proteins (Fig. 2e, bottom).

To visualize the proteomic changes occurring during the mission, we log transformed the R + 0/Pre ratios for each astronaut and compared them in a scatterplot (Fig. 2f). We could thus distinguish the proteins that had the same expression trend in both astronauts (quadrants I and III) from those that had opposite trends (quadrants II and IV). Fisher’s exact test revealed that basement membrane proteins were the highest enriched in astronaut A (green dots). Likewise, proteins with keratin annotations were the top enrichment among those with higher expression in astronaut B at R + 0 (red dots).

To explore the recovery phase after spaceflight, we used volcano plots to carry out pairwise comparisons between the biopsies taken at landing and those sampled two weeks after landing. In both astronauts there were many proteins showing differential abundance at these two time points (Supplementary Fig. 2g). Many annotations highly enriched at R + 0 were related to cell junctions and cytoskeleton (Supplementary Data 3). We also asked whether two weeks on Earth were sufficient for full recovery of the muscle of astronauts by comparing the biopsies taken at R + 14 to those from pre-mission. The large number of significant hits in this longitudinal comparison suggests that the muscle was in a process of re-adaptation two weeks after landing and was still different in many ways from the pre-flight basal condition (Supplementary Fig. 2h). Annotations related to cytoskeletal proteins were enriched in the biopsies pre-flight, suggesting incomplete recovery of myofibrillar proteins (Supplementary Data 4).

### Remodeling of the skeletal muscle mitochondrial proteome during a space mission

**Fig. 3:**
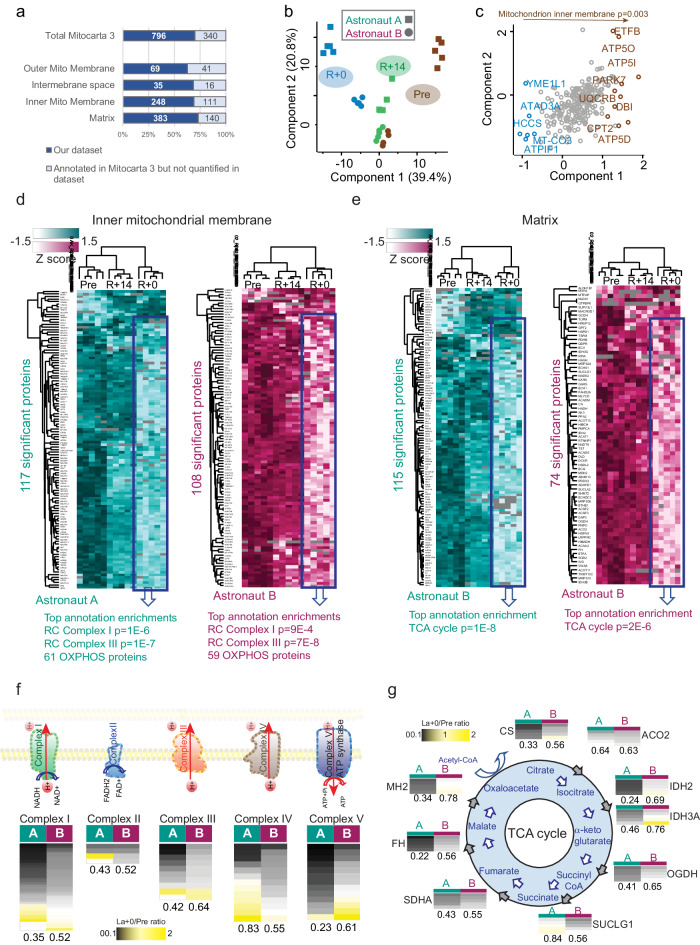
Mitochondrial proteome changes during spaceflight. **a** Coverage of mitochondrial proteome (top bar) and of different mitochondrial compartments in the dataset (based on MitoCarta3.0, see “Methods”). For each bar, 100% represents the total number of proteins annotated to each compartment in the human proteome human proteome (20591 entries), the blue segment shows the percent coverage in this dataset. **b** Principal component analysis (PCA) performed on 296 mitochondrial proteins that were expressed in all samples and technical replicates (*n* = 30), separating biopsies taken pre-mission (Pre, brown) from those taken on the day of landing (R + 0, blue) and 14 days after landing (R + 14, green). **c** Corresponding PCA loadings, with gene names of proteins driving the separation into component 1 highlighted in different colors for each timepoint. Highest annotation enrichment in component 1 is shown on top with corresponding *p* value. **d** Unsupervised hierarchical clustering of proteins of the inner mitochondrial membrane (from MitoCarta3.0) quantified in the muscle biopsies of the three timepoints of the mission (technical quintuplicates, *n* = 5 per astronaut). Proteins used for clustering (number on the left of the heat map) have significantly different expression differences in the biopsy pre-flight compared to the day of landing (two-sided paired Student *t* test, FDR = 0.05 for cutoff). The Z score color scale for each astronaut is shown on top left. Top annotation enrichments in clusters (indicated by a rectangle) are shown at the bottom of each heat map. **e** Same as D for mitochondrial matrix proteins. **f** Heat map of expression ratio in the two astronauts of all proteins quantified in the five complexes of the mitochondrial respiratory chain (schematic representation on top, ratio color scale at bottom). Each column shows the individual protein ratios in one astronaut (R + 0/Pre) as indicated above, with median ratio indicated at the bottom of each column. Values for each protein are median of 5 technical replicates. **g** Expression ratio of the enzymes catalyzing the reactions of the tricarboxylic (TCA) cycle in the two astronauts. The corresponding substrates are shown within the blue circles. For each enzyme, the median ratio (R + 0/Pre) and individual ratio values of technical replicates are shown. Ratio color scale top left.

Our data suggest a net decrease of mitochondrial protein expression during the space mission, in line with previous findings at the transcriptome level^36^. We thus used the MitoCarta3.0 database^50^ to annotate and select mitochondrial proteins from our dataset. This yielded a list of 796 quantified mitochondrial proteins, out of the total of 1136 in MitoCarta3.0, amounting to a coverage of 73%. We then used the database-associated submitochondrial location information to determine the coverage of different mitochondrial compartments, which ranged from 62% from the outer mitochondrial membrane (OMM), to 75% for the matrix (Fig. 3a). Unlike the whole muscle proteome, the MitoCarta3.0-derived mitochondrial proteome of both astronauts separated by PCA components 1 and 2 according to the time point of the mission. This suggests that mitochondrial remodeling in response to spaceflight was partially similar in the two subjects, despite a large individual variation detected at the muscle whole proteome level (Fig. 3b and see Supplementary Fig. 2a, b for comparison). Proteins of the mitochondrial inner membrane, such as complex V/ATPase subunits, were major drivers of the separation of muscle biopsy samples taken pre-mission from those taken on the day of landing (Fig. 3c).

Next, we set out to explore the proteomic changes occurring in the inner mitochondrial membrane. For each astronaut we selected the proteins that localize in this submitochondrial compartment and carried out two-sided paired Student *t* tests comparing biopsies taken pre mission (Pre) and on the day of landing (R + 0). Significant hits were then visualized by unsupervised hierarchical clustering. In both astronauts, this procedure highlighted a large cluster containing most hits, displaying a sharp decrease at R + 0. In both astronauts, this cluster was significantly enriched in proteins involved in oxidative phosphorylation (OXPHOS). The biopsy taken two weeks after landing (R + 14) clustered together with the biopsy sampled pre-mission, suggesting more similarity between these samples. The Earth environment thus seems to promote a rapid reversal of the effects of spaceflight at the level of inner mitochondrial membrane (Fig. 3d and Supplementary Data 5-6). To extend this finding to other mitochondrial compartments, we applied the same procedure to the proteins annotated to the matrix. The largest cluster of significant hits was expressed at much lower level at R + 0 than pre-mission and significantly enriched in tricarboxylic acid (TCA) cycle annotation in both astronauts. Proteins involved in fatty acid metabolism were also significantly enriched in the cluster displaying decreased intensity at R + 0. In the case of matrix proteins as well, the biopsy taken at R + 14 clustered with the biopsy taken pre-mission (Fig. 3e and Supplementary Data 7 and 8). We detailed the changes in OXPHOS proteins in each astronaut by quantifying the R + 0/Pre ratio for all subunits of the respiratory chain, subdivided by complex. In line with our previous results, the majority of OXPHOS complex subunits displayed a decrease at R + 0. The samples from the two subjects displayed generally the same trend, however the decrease in OXPHOS expression was generally larger for astronaut A (Fig. 3f). The expression ratio of the core components of the TCA cycle followed the same pattern as the respiratory chain subunit, namely (i) their expression was lower in the biopsy taken on the day of landing and (ii) astronaut A displayed in most cases larger decreases than astronaut B (Fig. 3g).

We carried out a similar analysis for proteins localized at the outer mitochondrial membrane. Paired *t* tests comparing the timepoints R + 0 and Pre yielded 11 and 21 significant hits for the two astronauts respectively. Most of these proteins decreased at R + 0 compared to both Pre and R + 14 timepoints. Among them were key players of mitochondrial dynamics, such as mitofusin 1 (MFN1) and mitochondrial fission protein1 (FIS1, Supplementary Fig. 3a). Direct comparison of the mitochondrial proteome of the two astronauts before the mission shows 242 proteins with significantly different expression. Apart from individual variability, this could be due to slightly different fiber type composition in the muscles of the two astronauts (see Fig. 1c). Of the proteins annotated to the respiratory chain and TCA cycle, 28 had significantly higher basal expression in astronaut A and 18 in astronaut B respectively, indicating comparable basal expression of proteins in these subcompartments alongside individual variability (Supplementary Fig. 3b). Our data thus point to a dramatic net decrease in volume and/or number of mitochondria in skeletal muscle in both astronauts. More intense training onboard of the ISS as in astronaut B correlated with some degree of preservation of key mitochondrial protein components during spaceflight.

### ROS detoxification and anti-oxidant response during spaceflight

**Fig. 4:**
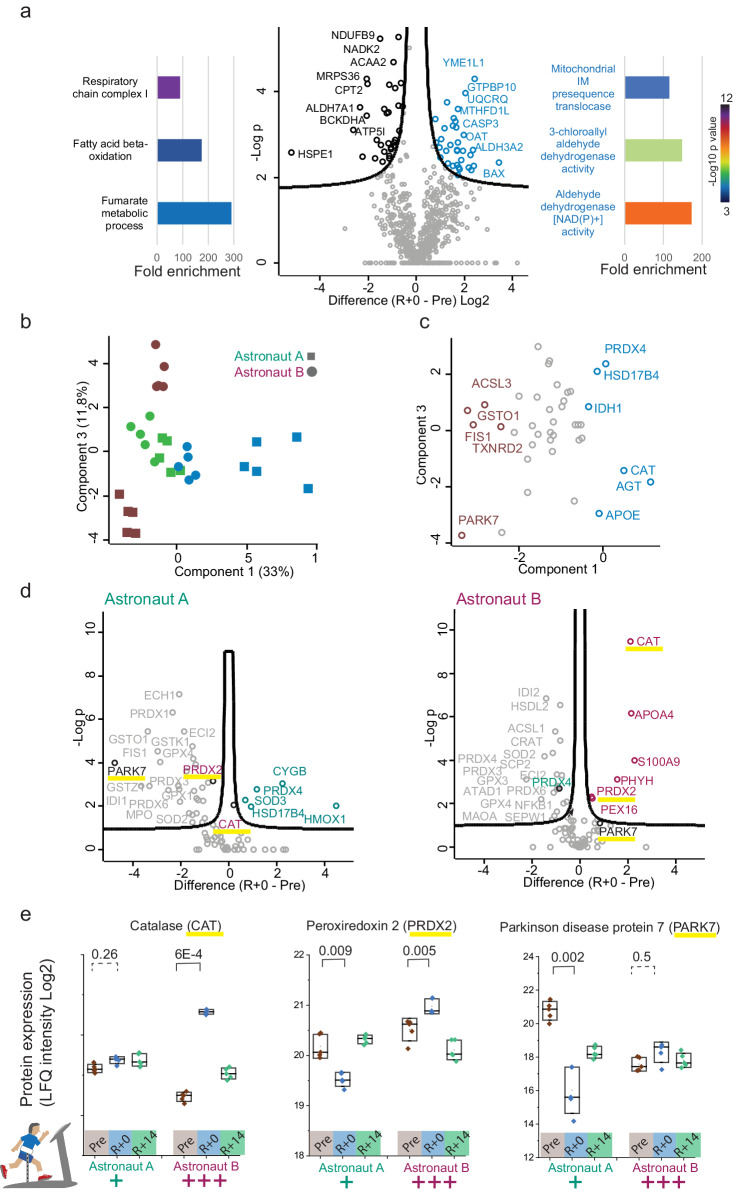
Regulation of anti-oxidant response and ROS scavengers during spaceflight. **a** Volcano plot of mitochondrial proteins (based on MitoCarta3.0) normalized by their summed intensity, comparing the biopsy taken pre-mission to that taken on the day of landing. Left side, highest annotation enrichments among proteins with higher normalized expression pre-mission (black). Right side, same analysis on the day of landing (blue). Background list, skeletal muscle proteome from the Human Protein Atlas (11756 entries). *P* value of the enrichment defines bar color, according to the scale on the right. **b** Principal component analysis on 109 proteins with antioxidant activity (GOMF, Keywords, Wiki pathways) in both astronauts (dot and square respectively) Different timepoints are shown in brown (Pre), blue (R + 0) and green (R + 14) as shown top right. **c** PCA loadings showing the proteins whose different expression between timepoints and subjects drive the separation into components. **d** Volcano plots of proteins with anti-oxidant activity (curated list of 109 proteins, see (**b**) and “Results” section) comparing the biopsies at timepoints R + 0 and Pre for astronaut A and B as indicated. Proteins underlined in yellow show large differences in the two individuals, see next panel. **e** Expression of three proteins with anti-oxidant activity showing different and/or opposite expression changes in the two astronauts. Box shows median expression, 25th and 75th percentile, whiskers show standard deviation. *P* value, two-sided paired *t* test. *n* = 5 technical replicates. Boxes are color-coded according to timepoint, as indicates in the legend within the plot, bottom.

To directly compare the different stages of the mission correcting for the decrease in the amounts of mitochondria, we normalized the expression of each MitoCarta3.0-selected protein by the summed expression of all mitochondrial proteins. Even after normalization, the expression of respiratory chain components and metabolic pathways located in the matrix, such as those involved in fatty acid oxidation, were significantly more abundant pre-mission than on the day of landing. (Fig. 4a, left panel). This suggests a more complex order of rearrangement during spaceflight than just a decrease in mitochondrial volume, possibly involving cristae remodeling programs. The protein most significantly overexpressed at R + 0 was YME1L1, an inner-membrane peptidase involved in promoting mitochondrial fusion. Proteins with higher normalized expression post-mission were enriched in annotations related to aldehyde dehydrogenase activity, which detoxifies aldehydic products generated by ROS (Fig. 4a, right side of volcano plot, Supplementary Data 9). Among them were 3-mercaptopyruvate sulfurtransferase (MPST) and Aldehyde dehydrogenase family 3 member A2 (ALDH3A2), an enzyme associated with ferroptosis in cancer^51^. Their expression at R + 0 was increased also prior to normalization (Supplementary Fig. 4a, b).

We then asked whether other features of a ROS detoxification response were affected in the muscle proteome during the space mission. We curated a list of proteins involved in the detoxification of reactive oxygen species by combining the annotations “peroxisome” (Keywords), “anti-oxidant” (gene ontology molecular function GOMF) with a literature-based list of mitochondrial ROS scavengers. This procedure resulted in a subset of 109 proteins (Supplementary Data 10) which yielded a separation along component 1 of principal component analysis according to the time of the mission for the biopsies of both astronauts (Fig. 4b, c). We then used volcano plots to compare the expression of these ROS scavengers in the Pre versus R + 0 muscle samples in each astronaut. We observed that most proteins with anti-oxidant activity in our list had higher expression in the biopsy pre-mission compared to that from the day of landing in both astronauts, suggesting a general down-sizing of the defense against ROS during spaceflight (Fig. 4d). Our data indicate a potentially dangerous mismatch between increased ROS production occurring in space^1,3^ and muscle ability to detoxify them.

Next, we asked whether the different training intensity of the two astronauts could influence their anti-oxidant response and ability of skeletal muscle to cope with proteostatic stress. In our dataset, we quantified fifty proteasome subunits and accessory proteins (Keywords annotation). In astronaut A, ten of them had significantly different expression comparing timepoints Pre and R + 0, four of which were upregulated and six downregulated at R + 0. In astronaut B, there were only two significant hits in the test, both of which decreased during spaceflight (Supplementary Fig. 4c). As for autophagy (Keywords annotation), five out of six significant hits were downregulated at R + 0 in astronaut A, whereas three out of five were upregulated in astronaut B. The catalytic subunit of 5’-AMP-activated protein kinase alpha-1 (PRKAA1) and protein S100-A8 had opposite expression trends in the two astronauts (Supplementary Fig. 4d, e). These latter results suggest that onboard physical activity may be impinging on the control of autophagy during spaceflight and at landing. We then focused on anti-oxidant enzymes with differential induction or downregulation in the two individuals. Astronaut B showed vigorous induction of catalase (CAT) during spaceflight, whereas only a minor increase could be measured in astronaut A (Fig. 4e). Interestingly, Peroxiredoxin 2 (PRDX2), a thiol-specific cytosolic peroxidase, displayed opposite changes in the two subjects, namely increasing during flight in astronaut B but decreasing in astronaut A (Fig. 4f). The expression of Parkinson disease protein 7 (PARK7), a stress sensor and redox-sensitive chaperone, was unchanged throughout the three phases of the mission in the muscle biopsies of astronaut B but it decreased over 90% at R + 0 for astronaut A (Fig. 4g). Interestingly, in the biopsies taken before the mission, astronaut A showed much higher expression of both PARK7 and CAT, whereas PRDX2 showed no expression difference (Supplementary Fig. 4f). Our dataset quantified 33 proteins annotated to the inflammatory response (Keywords annotation). Four and six of these had significantly different expression comparing Pre with R + 0 in the two astronauts respectively. In astronaut B, four out of six inflammatory markers were upregulated at R + 14 compared to Pre and R + 0, including the macrophage migration inhibitory factor (MIF), a pro-inflammatory cytokine (Supplementary Fig. 4g). These findings suggest that the regulation of the antioxidant response, the proteostatic network and inflammation during the mission may have been distinct for the two astronauts. A combination of genetics and different levels of exercise possibly underlies diverse outcomes.

## Discussion

Decades of aerospace medicine and biology have highlighted the consequences of microgravity and ionizing radiation, the major hazards threatening all organ systems of the human body during long spaceflight^1,52,53^. Mitochondrial stress and remodeling of gene expression related to energy metabolism are emerging as main consequences of spaceflight across multiple human tissues and animal models^36^.^3,54,55^. Together with atrophy and loss of force, this leads to strong skeletal muscle deconditioning which can only be partially attenuated by exercise countermeasures onboard. Ground-based models of muscle unloading, such as prolonged bed rest and dry immersion, cause muscle atrophy and decreased force production similarly to lack of gravity in space. On Earth, however, this is accompanied by only minor decreases in the expression of mitochondrial proteins^40,56^.

Here, we comprehensively reanalyzed our previously measured mass spectrometry-based proteomics dataset to detail the changes occurring in the muscle biopsies of two astronauts, taken before and after a six-months mission on the ISS. Astronaut B trained altogether 50% more than astronaut A (see Fig. 1b), which correlated with a more severe loss of fiber CSA and isometric force measured at landing in the latter^38^. Our proteomic dataset measured each muscle biopsy in quintuplicates. We focused our analysis on proteins and pathways that were changing their expression at different phases of the mission in both astronauts, thus limiting the effects of subject variability. Mitochondrial proteins were the most enriched among those downregulated in the biopsy from the day of landing compared to that taken pre-mission.

Our analysis reveals a dramatic decrease in the expression of mitochondrial proteins located in all main compartments of the organelle, particularly the inner mitochondrial membrane and the matrix. Despite their very different type of exercise, the two astronauts lost 43% and 55% of the total protein complement of their OXPHOS during the mission. It will be interesting to verify whether this downregulation occurs during spaceflight and whether it is dependent on exercise countermeasures. The expression of proteins located in the mitochondrial matrix, the intermembrane space and the outer mitochondrial membrane were also consistently decreased in both astronauts (see Fig. 3 and S3).

This relative lack of quantitative correlation between the amount of exercise countermeasures and the remodeling of the mitochondrial proteome during spaceflight prompted us to hypothesize that ionizing radiation and other sources of oxidative stress onboard, but not inactivity per se, may be the primary hit of skeletal mitochondrial damage in space. This is in line with data showing mitochondrial dysfunction during spaceflight in cell types that are not mechanically active and not expected to respond directly to activity, such as blood cells^3^. In this scenario, production of reactive oxygen and nitrogen species (ROS, RNS), would cause progressive mitochondrial damage, leading to increased mitophagy and net loss of mitochondrial protein expression. Our data show increase in the expression of redox proteins, such as ALDH3A2 and MPST, in the muscle sampled on the day of landing compared to the biopsy pre-mission. This finding confirms data from previous studies in larger cohorts of astronauts^36,57^. Although not measuring ROS production directly, our proteomic data suggest that oxidative damage may be “guilty by association” in the skeletal muscle remodeling occurring during long spaceflight. Defects in OXPHOS and electron transfer capacities, electron leak, H_2_O_2_ production, and the ratio between oxidative and antioxidant enzymes may be potentially responsible for the oxidative damage measured in different organisms and cell systems after spaceflight^58,59^. Our data also show significant expression regulation of proteins annotated to the inflammatory response at different phases of the mission, in line with the view that spaceflight is a prolonged stressor, linked to a proinflammatory status^48,60^. Interestingly, astronaut B showed higher upregulation of inflammatory markers than astronaut A during the reloading phase. Studies on hindlimb suspension in rats showed that 12.5 days of unloading cause degenerative and inflammatory changes, including muscle fiber damage and macrophage infiltration, which are not present immediately after reloading. These changes were detected as soon as 12 h after reloading and became more evident at 24 and 48 h^61^. In these astronauts, therefore, they may not yet be evident in the biopsies taken at R + 0 within 24 h of landing but ongoing at R + 14. Different amounts of physical activity in the reloading phase might determine the features of the ensuing inflammatory response and influence long term recovery postflight. This seems consistent with the observation that humans returning to Earth after spaceflight frequently reported muscle weakness and delayed-onset muscle soreness indicative of muscle cell damage^62^. This hypothesis is supported by findings on human single fibers^63^ and on whole muscle in-vivo after 17-day Space Shuttle flight^64^.

**Fig. 5:**
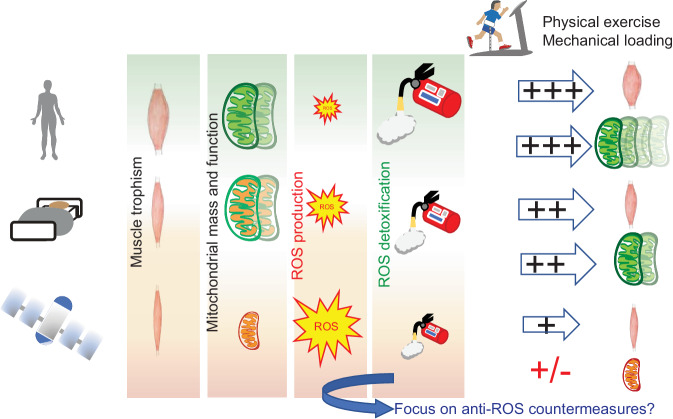
Exercise modulates the antioxidant response to preserve the mitochondrial proteome during spaceflight. Unloading and inactivity are serious challenges for skeletal muscle during long missions in space and a major risk factor in future manned missions to Mars. However, the basic cause-effect relationship between physical exercise, muscle growth and mitochondrial biogenesis is lost in the space environment. We hypothesize that reactive oxygen species (ROS), possibly derived from ionizing radiation, may be the primary hit of skeletal mitochondrial damage in space. Physical exercise might have a mitigating effect, possibly through upregulation of anti-oxidant responses (represented as a fire extinguisher).

Our data show a positive correlation between higher amount of exercise and the expression of key autophagy players. In addition, induction of catalase, a key dismutase involved in the detoxification of hydrogen peroxide^65^ may be under partial control of physical activity. Indeed, while astronaut B had over four-fold induction of this protein at R + 0 compared to pre-mission, astronaut A had negligible expression changes. Other enzymes involved in the cell antioxidant response, such as peroxiredoxin 2, had a pattern of induction at R + 0 that correlated with the different levels of exercise (see Fig. 4). Given the established role of exercise in the regulation of redox signaling on Earth^66^, we propose that the partial protective effect of exercise on the mitochondrial proteome in space might be modulated by the anti-oxidant response in skeletal muscle (Fig. 5). Physical activity on Earth causes muscle growth and mitochondrial biogenesis, and triggers a physiological balance of ROS generation and antioxidant responses that are fundamental features of muscle health ad performance^67^. Inactivity on Earth triggers rapid muscle atrophy^68^ but minor mitochondrial changes in the same timeframe^39^, suggesting that ROS production is low and/or efficiently counteracted by cellular defenses. During spaceflight, however, increased oxidative stress might overpower the cellular capacity for detoxification, leading to mitochondrial damage and loss of OXPHOS capacity. Given the tight relationship between the integrity of the mitochondrial network and the preservation of muscle mass^69^, this might further contribute to the functional decline of skeletal muscle in spaceflight. Oxidative damage would be parallel and independent of the direct effects of unloading, explaining the scarce efficacy of exercise countermeasures. Recent advances in exercise protocols aimed specifically at astronauts targeted different muscles and monitored the divergent responses to atrophy of soleus and vastus lateralis^70^. In addition, the relationship between exercise countermeasures during bed rest and mitochondrial respiration was investigated in peripheral blood mononuclear cells^71^. This type of studies will be instrumental to advance readouts to monitor mitochondrial health and progress countermeasures during spaceflight. Whether our findings on soleus muscle can be extended to other lower limb muscles is uncertain, as there is clear diversity in the response to disuse^72,26^. Soleus has a higher content of type1-slow fibers, and thus contains more mitochondria and likely produces more ROS than gastrocnemius and vastus lateralis^73^. While mitochondria are not the main source of ROS production during muscle contraction, their contribution becomes prevalent during inactivity^74^. The shift from postural tonic activity to inactivity in microgravity is thus expected to affect soleus more than faster muscles. However, we have previously shown that slow fibers may be better equipped for the defense against ROS through efficient generation of NADPH^75^. Moreover, human muscle have a slower phenotype than muscles of small rodents and the difference in mitochondrial content between slow and fast human fibers is much lower than in mouse muscles, as shown by comparing histochemical staining for succinate dehydrogenase in the two species^76^.

We are aware of several limitations of this study: (i) first and foremost, it is based on two subjects only and does not provide orthogonal validations, as the residual biopsy material was used up in 2022 to produce our proteomic dataset. Many effects of spaceflight on the human body display large individual variability^77^ and further studies with more astronauts will be necessary to strengthen the data reported here. In addition, changes in skeletal muscle of women after long mission on the ISS should be systematically assessed, as many organs display sex differences in the response to oxidative stress^78^; (ii) a further consequence of the analysis of two astronauts only and one muscle biopsy per timepoint is that statistics in our proteomic dataset are based on repeated MS measurements of each muscle biopsy. This reinforces the need to put our interpretation into perspective using larger cohorts of astronauts; (iii) during the first hours after landing and before the muscle biopsies were taken, the astronauts could move freely at Earth gravity and their activity was not monitored. Distinguishing cumulative effects due to spaceflight from early effects of reloading after landing is thus not possible with this experimental design.

Our present effort aimed at extracting further information from these unique samples. Based on this reanalysis, we suggest that the search for countermeasures meant to preserve muscle performance during spaceflight should consider the antioxidant response as a primary target and exploit its mechanistic correlation to physical activity.

## Methods

### Previous analysis

The mass spectrometry-based proteomics data analyzed in this paper have been generated as part of the experimental design of our previous paper entitled Signatures of muscle disuse in spaceflight and bed rest revealed by single muscle fiber proteomics, by M. Murgia et al.^39^, where limited analysis of these data can be found. Likewise, the proteomic data were deposited to the ProteomeXchange Consortium via the PRIDE partner repository with the dataset identifier PXD028435 and are publicly available. All methods are detailed in that publication and briefly reported here.

### Study subjects

Two subjects of equal sex and comparable age, were recruited for a half-year mission on the International Space Station. All procedures were performed in accordance with the guidelines and approved by the Committee for the Protection of Human Subjects (NASA MPA number 7116301606HR, Protocol number 09-3940-Ren-2-Air-1). Written informed consent was obtained from the two subjects prior to study inclusion. Muscle biopsies were collected pre-flight (day -79 and -76 for the two subjects respectively), on the day of landing (R + 0) and two weeks after landing (R + 14). Subjects were walking but not exercising on the day of landing. The R + 0 biopsy was taken around 24 h after landing, as the astronauts had to fly to another location for physical examination and sample collection. We refer to the time of biopsy collection as R + 0, whereas another study defined it as R + 1. Likewise, we use R + 14, corresponding to their R + 15.

### Muscle lysate sample processing

Muscle lysates of astronauts in RIPA buffer were precipitated in acetone and resuspended in LYSE buffer (PreOmics). After overnight digestion the lysate was desalted and loaded onto StageTip plugs of SDB-RPS. Purified peptides were eluted with 80% acetonitrile-1% ammonia and dried. The dataset consists of technical quintuplicates for the biopsies of 2 astronauts at 3 time points.

### Liquid chromatography and mass spectrometry

Peptides were separated on 50 cm columns of ReproSil-Pur C18-AQ 1.9 μm resin (Dr. Maisch GmbH) packed in-house. Liquid chromatography-mass spectrometry (LC-MS) analysis was carried out on an anEASY-nLC-1200 system (Thermo Fisher Scientific) connected to a trapped ion mobility spectrometry quadrupole time-of-flight mass spectrometer (timsTOF Pro, Bruker Daltonik) using ddaPASEF^49^.

### Computational proteomics

The MaxQuant software (version 1.6.15.0)^79^ was used for the analysis of raw files searching against the human Uniprot databases (UP000005640_9606, UP000005640_9606_additional) and a common contaminants database. The false discovery rate (FDR) was set to 1% for peptides (minimum length of 7 amino acids) and proteins and was determined by searching a reverse database. Peptide identifications by MS/MS were transferred by matching between the runs with a 0.7-min retention-time match window between all sample from the original dataset (single fibers and libraries). Peptides with a minimum length of seven amino acids were considered for the search including N-terminal acetylation and methionine oxidation as variable modifications and cysteine carbamidomethylation as fixed modification. Enzyme specificity was set to trypsin cleaving c-terminal to arginine and lysine. A maximum of two missed cleavages was allowed. For MYH isoforms specifically, only peptides unique to each isoform were used for quantification in MaxQuant.

### Bioinformatic and statistical analysis

Analyses were performed with the Perseus software (version 1.6.15.0), part of the MaxQuant environment^80^. Label free quantification (LFQ) values were used throughout the analysis for protein expression, using the feature implemented in MaxQuant. Proteins from a list of common contaminants were not deleted from this dataset and the “contaminant” label can be found in the Protein groups columns, as some of them are human cytoskeletal and membrane proteins expressed in human muscle. Categorical annotations were supplied in the form of UniProt Keywords, Corum, KEGG and Gene Ontology. Annotation enrichments were calculated by Fisher exact test using 0.02 Benjamini-Hochberg FDR for truncation and the muscle tissue map of the Human Protein Atlas containing 11756 entries as background^81^. Where indicated, proteins of plasma and erythrocyte origin were excluded from the background, based on the corresponding lists provided by the Human Protein Atlas^81^. Principal Component Analysis was based on common proteins, detected in both astronauts. We first calculated the median expression of five technical (LC-MS) replicates per biopsy and then discarded away proteins with fewer than 6 valid values.

In this dataset, technical variance is the variance between LC-MS replicates, and biological variance is the variance between two muscle biopsies. Our data show a high degree of reproducibility between technical replicates, based on Pearson’s rho (See Supplementary Fig. 1c). This is part of the Perseus workflow and applies this formula:

1 $$r=\frac{\sum(x_{i}-\bar{x})(y_{i}-\bar{y})}{\sqrt{\sum(x_{i}-\bar{x})^{2}(y_{i}-\bar{y})^{2}}}$$

Pairwise correlations are then carried out between all data columns.

Based on these calculation, technical variance was consistently lower than the variance between biological classes, i.e muscle biopsies (see Supplementary Fig. 1c). We thus applied a statistical strategy based on the two-sided paired Student’s *t* test to compare protein expression levels in biopsies from different timepoints of the same astronauts.

We compared the list of proteins whose expression changed significantly in both astronauts between two timepoints using Venn diagrams (as in Fig. 2c, e). Common proteins, at the intersection, were further analyzed. *P* values were determined using an exact approach and False Discovery Rate (FDR) was used to control for multiple comparisons (significance threshold set at FDR < 5%).

For the volcano plots the dataset was filtered for 3 valid values in at least one group (one biopsy and 5 technical replicates). P-values were calculated for each protein comparison using the *t* test formula and then adjusted to a permutation-based false discovery rate of 5%. We set an S0 parameter at 0.1 to account for effect size in our significance threshold. Unsupervised hierarchical clustering was applied based on Euclidean distance after Z-scoring. MitoCarta3.0^50^, containing a curated inventory of 1136 human mitochondrial genes, was used to assign mitochondrial localization to the proteins quantified in our dataset. Likewise, the summed intensities of these proteins were used for the normalization applied in Fig. 4a.

### Reporting summary

Further information on research design is available in the Nature Research Reporting Summary linked to this article.

### Supplementary information

Supplemental Information

Reporting Summary

Supplementary Data 1

Supplementary Data 2

Supplementary Data 3

Supplementary Data 4

Supplementary Data 5

Supplementary Data 6

Supplementary Data 7

Supplementary Data 8

Supplementary Data 9

Supplementary Data 10

## Data Availability

The mass spectrometry-based proteomics dataset analyzed during the current study is available in the ProteomeXchange repository with the dataset identifier PXD028435.
