# Supplementary material for: Spaceflight on the ISS changed the skeletal muscle proteome of two astronauts
Source: NPJ Microgravity. 2024 Jun 5;10:60. doi: 10.1038/s41526-024-00406-3 (PMC11153545; doi:10.1038/s41526-024-00406-3)
Supplement: Supplementary file 1 — Supplemental Information [file 41526_2024_406_MOESM1_ESM.pdf]

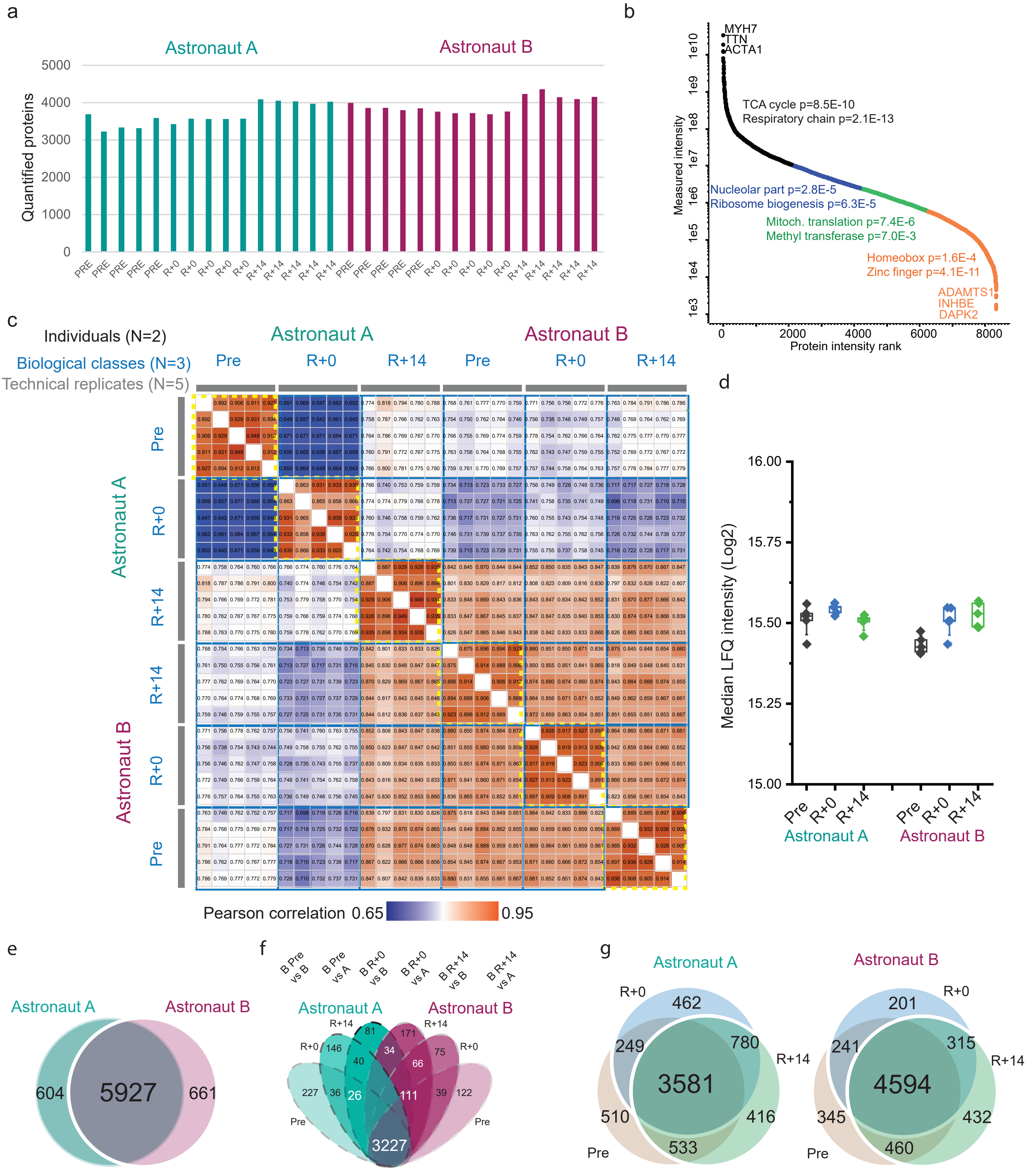

Supplementary Figure 1. Proteomic features of the dataset

(a) Number of quantified proteins in individual MS runs for the two astronauts as indicated. **(b)** Intensity distribution of the 7146 proteins quantified in the dataset, divided in quartiles (see different symbol color). The top protein annotation enrichments in each quartile are shown next to the curve with the corresponding p value (Fisher's exact test, FDR 0.02). The gene name of three most and least intense proteins is shown on top and bottom of the curve respectively. **(c)** Matrix of Pearson correlations between label-free quantification (LFQ) intensities of all muscle biopsy samples in the dataset, comprising 2 astronauts, 3 biological classes (biopsies taken Pre, R+0 and R+14 as indicated), and 5 technical replicates. The dashed yellow squares in the middle of the plot highlight the correlation between technical replicates, the blue square show correlation between biological classes (different biopsies). Numeric values of Pearson correlation are shown for each comparison of 30 samples and colored according to the scale at the bottom for reference. **(d)** Median intensities (Log2) of all proteins quantified in each sample (total number of quantified proteins varied between 3201 and 4342 in different samples). Each box shown the median, 25th and 75th percentile of n=5 technical replicates, shown as individual da points. Whiskers represent standard deviation. **(e)** Total overlap of proteome quantification between the two astronauts visualized as Venn diagram. For each astronaut, we used the total protein quantification of three muscle biopsies, each in technical quintuplicates. In the respective areas, number of proteins common to both astronauts and exclusively quantified in one of them. **(f)** Overlap of proteome quantification across different time points of two astronauts. The number of proteins exclusively quantified in one time point and common to two and three timepoints of each astronaut are shown. **(g)** Overlap of proteome quantification in the three biopsies of each astronaut at different timepoints. For f and g, each timepoint shows the total protein quantification in five technical replicates of a muscle biopsy.

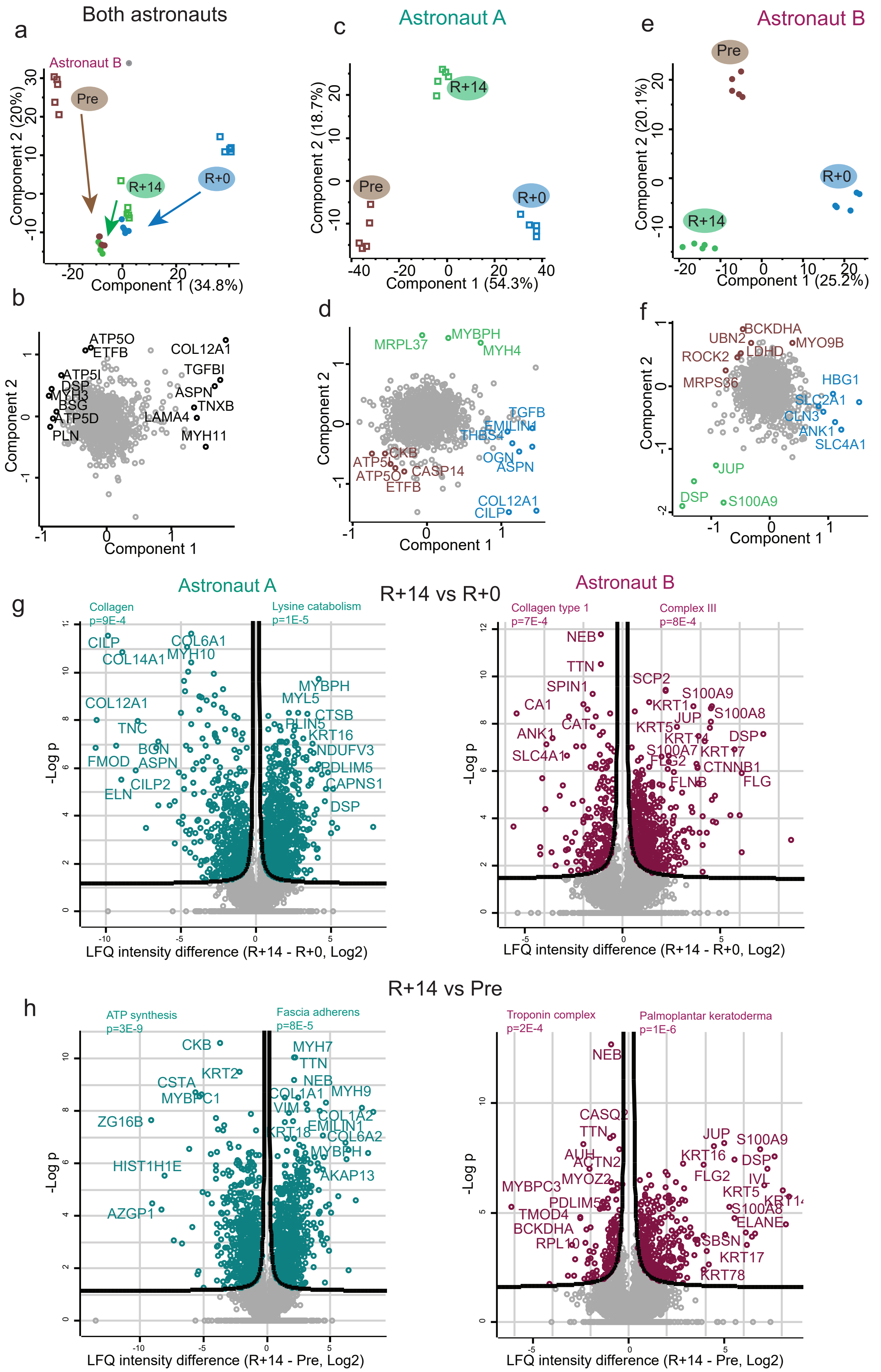

Supplementary Figure 2. Comparison of muscle biopsies at different time points of spaceflight

(a) Principal component analysis (PCA) of replicates of six muscle biopsies of two astronauts at timepoints Pre (brown), R+0 (blue) and R+14 (green) **(b)** PCA loadings corresponding to A. **(c)** PCA for astronaut A only. **(d)** PCA loadings for astronaut A only. **(e)** PCA for astronaut B only. **(f)** PCA loadings for astronaut B only. **(G)** Volcano plots comparing the biopsies taken at R+14 with that taken at R+0 for astronaut A (left panel) and astronaut B (right panel). N=5 technical quintuplicates. **(h)** Same analysis comparing the biopsies taken at R+14 with those pre-mission for astronaut A (left panel) and astronaut B (right panel). Top annotation enrichments on each side of the volcano plots are indicated, with corresponding p values (Fisher exact test, FDR=0.04). Label-free quantification (LFQ) intensity quantifies protein abundance (see Methods).

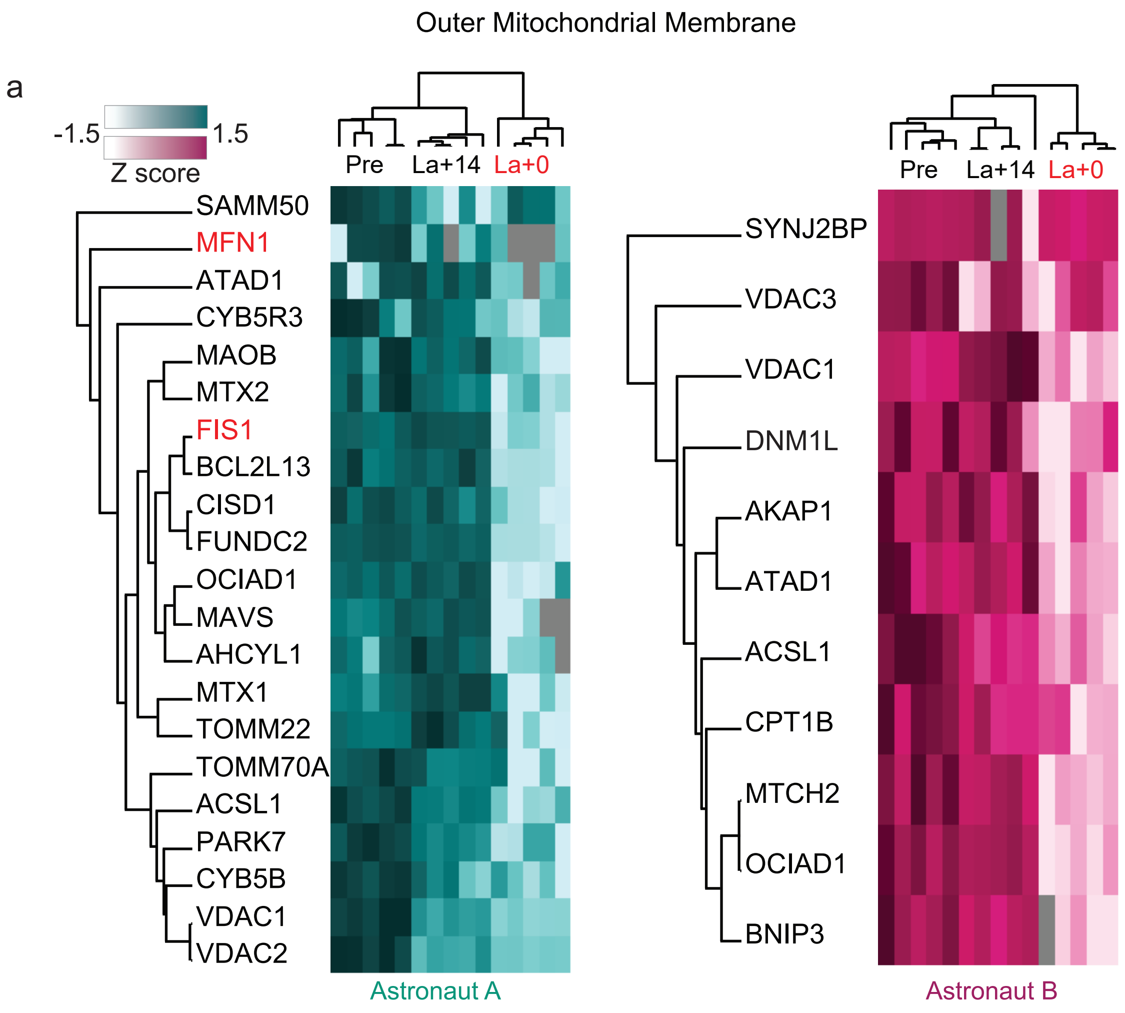

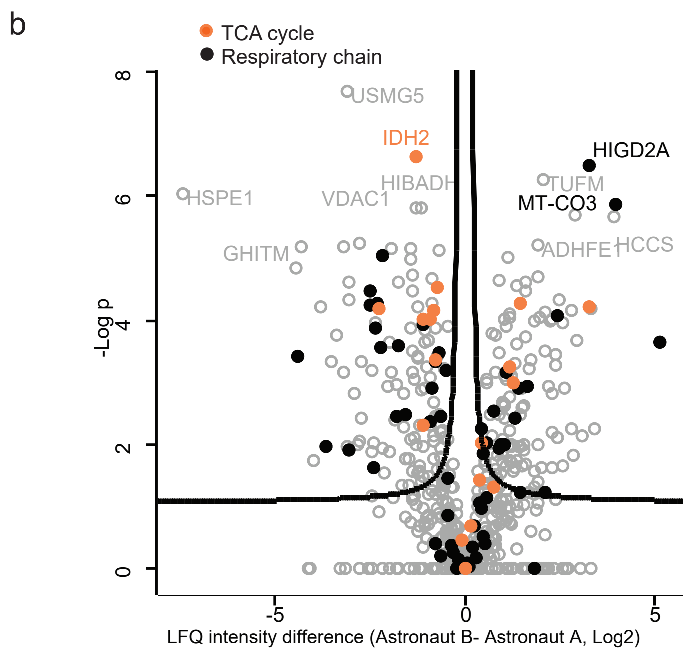

Supplementary Figure 3. Comparison of the mitochondrial proteome of the two astronauts.

(a) Unsupervised hierarchical clustering of proteins of the outer mitochondrial membrane (from MitoCarta3.0) quantified in the muscle biopsies of the three timepoints of the mission Proteins used for clustering have significantly different expression differences in the biopsy pre-flight compared to the day of landing (paired Student T-test, FDR=0.05 for cutoff, technical quintuplicates, N=5 per astronaut). Proteins involved in mitochondrial fission and fusion are marked in red font. Corresponding Z score scale, top left. **(b)** Volcano plot comparing the mitochondrial proteome (from MitoCarta3, 796 quantified proteins) of the two astronauts before the mission. No preferential distribution of tricarboxylic (TCA) cycle (orange dots) and respiratory chain annotations (black dots) is observed. Label-free quantification (LFQ) intensity quantifies protein abundance (see Methods).

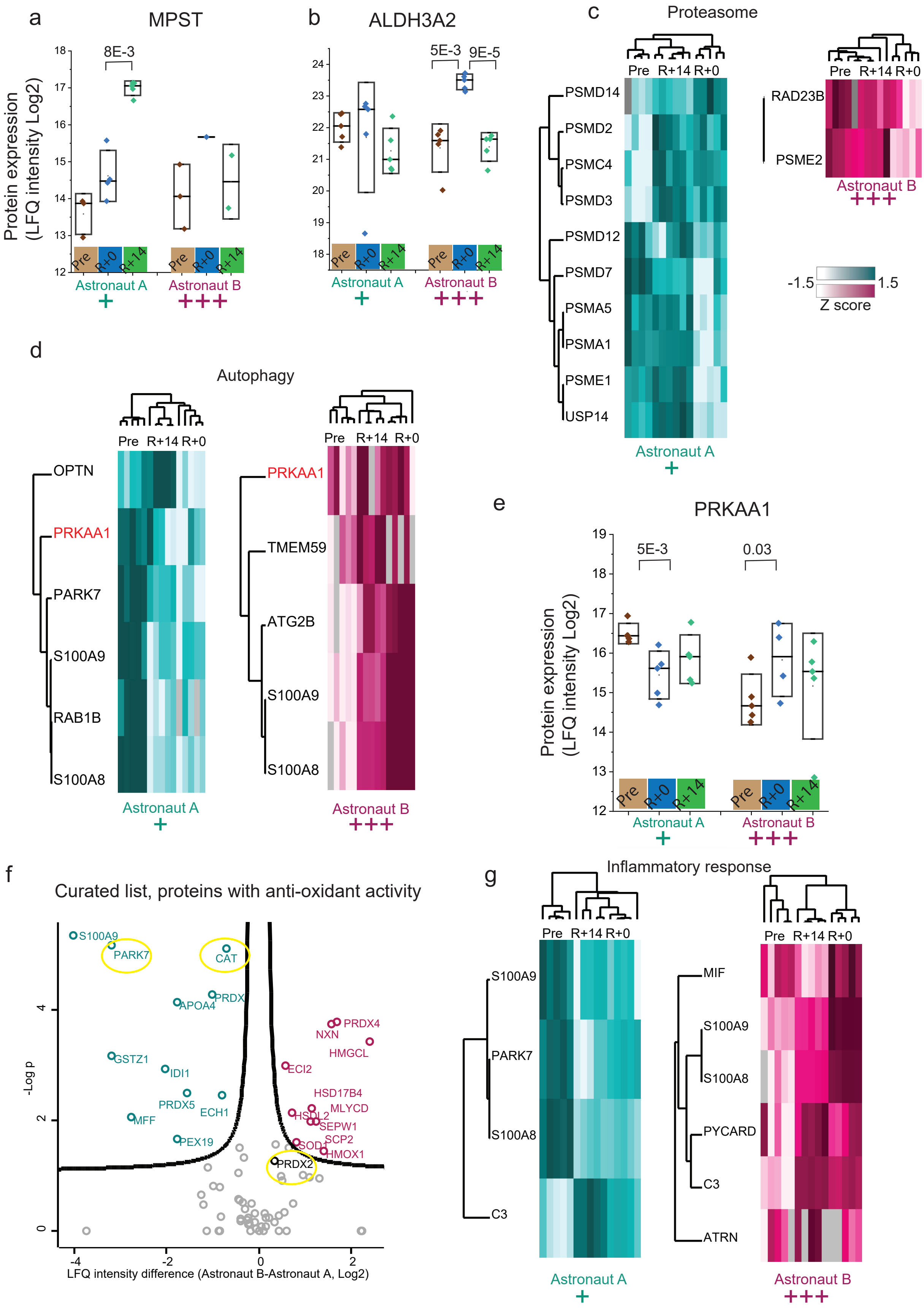

Supplementary Figure 4. **Anti-oxidant, proteasome, autophagy and inflammation repertoire in the two astronauts at different phases of the mission.**

(a, b) Expression of 3-mercaptopyruvate sulfurtransferase (MPST) and Aldehyde dehydrogenase family 3 member A2 (ALDH3A2), two enzymes detoxifying aldehydic products, in the two astronauts at different timepoints. Box shows median expression, 25th and 75th percentile, whiskers show standard deviation. P value, paired t-test. N=5 replicates. **(c)** Unsupervised hierarchical clustering of proteasome subunits (Keyword annotation Proteasome) for each astronaut. Proteins used for clustering have significantly different expression differences in the biopsy pre-flight compared to the day of landing (paired Student T-test, FDR=0.05 for cutoff). **(d)** same as c for proteins involved in autophagy (Keyword annotation). (e) Expression of 5'-AMP-activated protein kinase catalytic subunit alpha-1 (PRKAA) in the two astronauts at different timepoints (see a,b). **(f)** Volcano plot comparing the expression of 109 antioxidant enzymes (see Figure 4 and Table S8) in the two astronauts before the mission. Three proteins whose expression at different phases of the mission are detailed in Figure 4e are circled in yellow. **(g)** same as c for proteins involved in inflammatory response (Keywords annotation). Label-free quantification (LFQ) intensity quantifies protein abundance (see Methods). + and +++ refer to the amount of onboard exercise of each astronaut (see Figure 1).
